# Classification, Naming and Evolutionary History of Glycosyltransferases from Sequenced Green and Red Algal Genomes

**DOI:** 10.1371/journal.pone.0076511

**Published:** 2013-10-16

**Authors:** Peter Ulvskov, Dionisio Soares Paiva, David Domozych, Jesper Harholt

**Affiliations:** 1 Department of Plant and Environmental Sciences, University of Copenhagen, Frederiksberg C, Denmark; 2 Department of Biology and Skidmore Microscopy Imaging Center, Skidmore College, Saratoga Springs, New York, United States of America; Lawrence Berkeley National Laboratory, United States of America

## Abstract

The Archaeplastida consists of three lineages, Rhodophyta, Virideplantae and Glaucophyta. The extracellular matrix of most members of the Rhodophyta and Viridiplantae consists of carbohydrate-based or a highly glycosylated protein-based cell wall while the Glaucophyte covering is poorly resolved. In order to elucidate possible evolutionary links between the three advanced lineages in Archaeplastida, a genomic analysis was initiated. Fully sequenced genomes from the Rhodophyta and Virideplantae and the well-defined CAZy database on glycosyltransferases were included in the analysis. The number of glycosyltransferases found in the Rhodophyta and Chlorophyta are generally much lower then in land plants (Embryophyta). Three specific features exhibited by land plants increase the number of glycosyltransferases in their genomes: (1) cell wall biosynthesis, the more complex land plant cell walls require a larger number of glycosyltransferases for biosynthesis, (2) a richer set of protein glycosylation, and (3) glycosylation of secondary metabolites, demonstrated by a large proportion of family GT1 being involved in secondary metabolite biosynthesis. In a comparative analysis of polysaccharide biosynthesis amongst the taxa of this study, clear distinctions or similarities were observed in (1) N-linked protein glycosylation, i.e., Chlorophyta has different mannosylation and glucosylation patterns, (2) GPI anchor biosynthesis, which is apparently missing in the Rhodophyta and truncated in the Chlorophyta, (3) cell wall biosynthesis, where the land plants have unique cell wall related polymers not found in green and red algae, and (4) *O*-linked glycosylation where comprehensive orthology was observed in glycosylation between the Chlorophyta and land plants but not between the target proteins.

## Introduction

The current proliferation of genomic sequencing studies with vascular plants has focused on species with economic significance or usefulness as model species. However recently, the selection of some taxa has been based on their presumed evolutionary significance with *Selaginella moellendorffii* [1], an extant representative of early vascular plants, as the prime example. Modern genomic analyses have also focused on lineages derived from the most primitive of photosynthetic eukaryotes, i.e., the algae, and are providing new and valuable insight into the evolution of life on the planet. For example, it is widely accepted that modern day plants evolved from green algal ancestors. Recent transcriptomic evidence [2], [3] has not only supported this but has also refined the identification of the specific extant taxonomic groups of green algae most closely related to modern day land plants. Likewise, genetic information has helped show that green and red algae represent two major groups of algae derived from the most primitive algal lineages and that from these organisms, emerged all other algal groups via secondary and tertiary endosymbiosis. Clearly, as more genomic data of algae is compiled and analyzed, more insight will be gathered concerning the evolution and processes of life.

**Figure 1:**
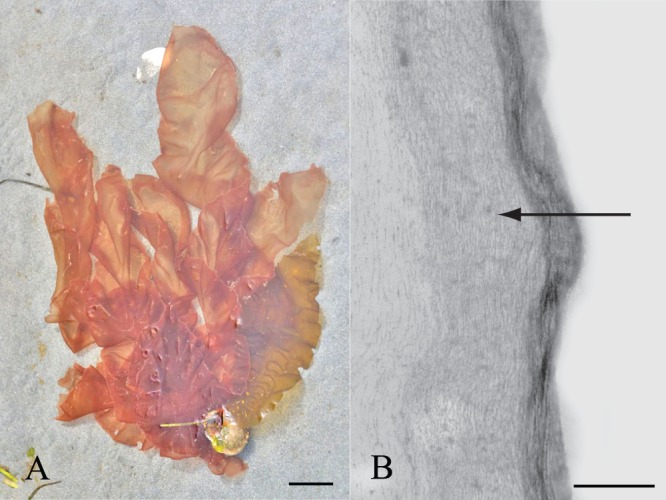
The red algal ECM. The ECM of red algae consists of fibrillar components and gel-like polysaccharides. (A) *Porphyra* is a common sheet-like red alga found in coastal waters throughout the world. Scale bar = 5 cm. (B) The walls of *Polysiphonia* are multilayered consisting of alternating layers of fibrils. Scale bar = 200 nm.

**Figure 2:**
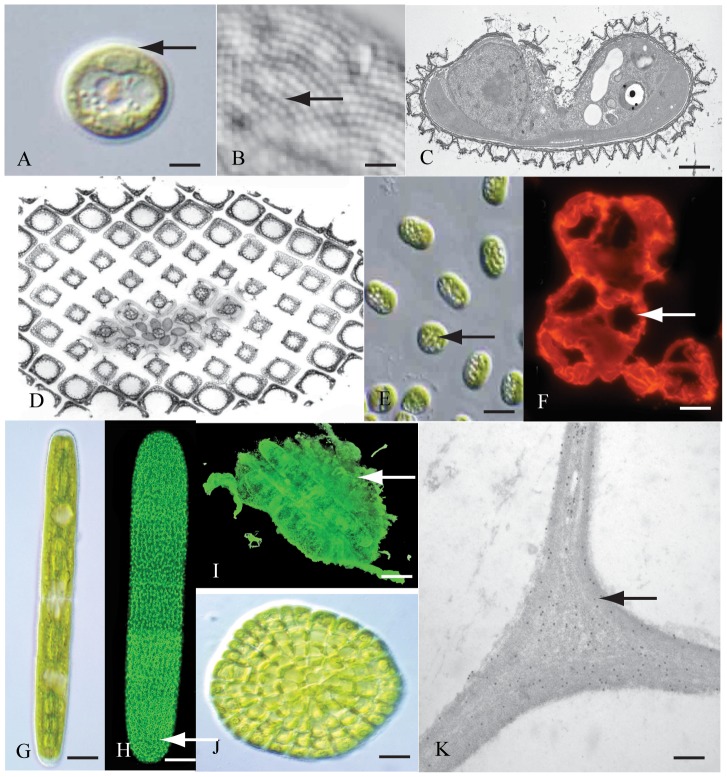
Variation in extracellular coverings of green algae. (A) *Mesostigma viride* (CGA) and many members of the Prasinophyceae (Chlorophyta) have a very distinct extracellular matrix (ECM; arrow). DIC image. Scale bar = 5 µm. (B) Close examination of the *Mesostigma* ECM reveals regular repeating units (arrow). DIC image. Scale bar = 175 nm. (C) The repeating units of the ECM represent the large body scales aligned upon the outer surface. TEM image. Scale bar = 100 nm. (D) In addition to the large outer body scales, two other body scale layers (arrow) are part of the inner regions of the ECM. TEM image. Scale bar = 50 nm. (E) In *Chlorokybus* (CGA), the ECM consists of a gel-like wall that holds cells together in sarcinoid packets (arrow). DIC image. Scale bar = 25µm. (F) The *Chlorokybus* sheath labels with JIM13 (arrow), a mAb with specificity toward arabinogalactan protein epitopes of land plants. Fluorescence microscopy. Scale bar = 30 µm. (G) In desmids like *Penium* (CGA), the ECM consists of a cell wall and extracellular polymeric substance or EPS. DIC image. Scale bar = 8 µm. (H) *Penium*’s cell wall is highlighted by a pectin-rich outer wall layer (arrow) as highlighted by JIM5 labeling. Fluorescence image. Scale bar = 4.5 µm. (I) The EPS of *Penium* is extensive as it covers the surface of cells after they stop gliding (arrow). The EPS was labeled with an anti-EPS antibody. Fluorescence image. Scale bar = 17 µm. (J) Thalloid CGA like *Coleochaete* have cell walls. DIC image. Scale bar = 25 µm. (K) The cell wall of *Coleochaete* also contains epitopes of wall polysaccharides that are similar to those found in land plants. Here, JIM5, with specificity toward pectin, labels the junction zone of three cells (arrow). Scale bar = 75 nm.

Green algae (Chlorophyta and Streptophyta: Viridiplantae) and red algae (Rhodophyta) represent modern assemblages of oxygenic photosynthetic eukaryotes derived from a heterotrophic ancestor whose plastid was derived via primary endosymbiosis approximately 1.5 billion years ago [4], [5]. Green and red algae, along with a third small taxon, the Glaucophyta, are allied by their primitive origin and differ distinctly from other lineages of photosynthetic eukaryotes, i.e., modern day “algae”, whose plastids are products of secondary or tertiary endosymbiosis (e.g., brown algae, diatoms; [6], [7]). Extant green and red algae exhibit remarkably diverse morphologies ranging from simple unicells through filaments to complex 3-dimensional thalli [2], [8] and have successfully exploited virtually every photic habitat. Several taxa of green algae have also successfully adapted to terrestrial ecosystems and in one case, yielded modern plants [8]. Both red and green algae have evolved well-developed carbohydrate metabolic pathways that encompass the ability to synthesize storage polyglucans (i.e., “starches”) via photosynthesis and to manufacture extensive extracellular matrices that consist of structurally-complex, carbohydrate-rich cell walls and exuded mucilages or slimes [9]. Storage polyglucans and extracellular matrix polysaccharides and glycoproteins are profoundly important. For example, the extracellular matrix, primarily consisting of complex and diverse polysaccharides, proteoglycans and glycoproteins, is vital to survival. It functions in such roles as physical and chemical defense, anti-desiccation, absorption and expansion. In red algae, cell wall-derived mucilages (e.g., agar, carrageenan) ensheath and protect cells in harsh saline habitats including intertidal zones (Figure 1). Many of these carbohydrates are also economically valuable especially in food, pharmaceutical and biofuels industries [10]. In green algae, diverse types of extracellular matrices allow for survival in such ecosystems as desert soils, tree bark, snow banks as well as marine and freshwater ecosystems (Figure 2). Adaptations in the cell wall of some green algae 450 million years ago also became instrumental in the invasion onto land and the subsequent evolution of land plants.

As more genomic sequencing data derived from red and green algae become available, the biosynthesis of carbohydrates, (i.e., glycome) will be a major focus of study for both the aforementioned reasons as well as for the importance of algal polysaccharides in applied technologies. Before this happens, it will be very useful if the system of classification of genes encoding glycosyltransferases (GTs) is organized and named in a consistent manner by both phycologists and plant biologists. In this paper, we present a new system of glycosyl transferase classification that is systematic, efficient and integrates effectively with what is currently used for land plants [1], [11]. This system will significantly aid in the correct identification of orthology and ultimately, support the construction of accurate evolutionary inferences for both red and green algae. Also, comparative genomic analyses of green algae and plants, as tools for elucidating functions as suggested by Hicks et al. [12] would benefit from a consistent classification and naming convention. This, in turn, will facilitate the elucidation of critical aspects of glycome biosynthetic adaptations during the evolution of land plants.

## Materials and Methods

### Proteomes and Database Creation

The filtered models proteome of *Chlamydomonas reinhardtii* P.A. Dangeard, *Volvox carteri f. nagariensis* M.O.P. Iyengar, *Cyanidioschyzon merolae* and *Galdieria sulphuraria* were acquired from Joint Genome Institute, CA, USA in the case of *C. reinhardtii* and *V. carteri f. nagariensis* and from http://merolae.biol.s.u-tokyo.ac.jp/and http://genomics.msu.edu/galdieria/index.html, for *C. merolae* and *G. sulphuraria*, respectively. *Arabidopsis thaliana*, *Oryza sativa* ssp. *japonica*, *Micromonas* sp., *Ostreococcus tauri* C. Courties & M.-J. Chrétiennot-Dinet and *Ostreococcus lucimarinus* CAZyome were acquired from CAZy and used as is. *S. moellendorffii* and the moss *Physcomitrella patens* CAZyome were acquired from Genbank using Harholt et al. [13] as a guide. Protein sequences of the CAZy-database were downloaded from Genbank and used for generating a CAZy-BLAST database. GTs from *Selaginella moellendorffii* were added manually as were genes with a DUF266 domain before generating a CAZy-BLAST database. Our analysis represents the state in CAZy as of May 2012. Sequences of GTs identified in the screen are available upon request and identifiers can also be found in Table S1.

### The Screen

The screen was performed as for *Brachypodium distachyon* [11], *S. moellendorffii* [1] and described in detail in Harholt et al. [13], in brief: Proteome files of each taxon were used to BLAST against the local CAZy-database using an e-value of 10^−25^ as a cut-off. Quality control comprised both batch-wise and manual steps. Hit sequences were rpsblasted against the conserved domain database (CDD; [14]. Sequences were eliminated where all significant CDs were incompatible with GT function. The CDD did not cover all CAZy-families and in these cases, the Phyre2 fold prediction server was used [15]. One significant fold match would pass the hit sequence to the manual quality control, inspection of the alignments, where false positives were eliminated. We have attempted not to give names to obvious fragments and pseudogenes, so the final threshold to pass was based on manual screening of alignments of genomes of interest.

### False Negatives

The unfiltered all protein models comprise models that are promoted to best models; models that are alternative models for the same genes; and models that have no homologs in best models. The third category was used for estimating the number of GT candidates that were missed by the screen, i.e., the false negatives. In the *Chlamydomonas reinhardtii* genome v.4 proteome all models were used in the analysis. Unique all models hits were defined as sequences for which a matching model (e-value <1×10^−40^) could not be found in best models. These were then analyzed by the main screen as described above, including validation of putative hits.

### Phylogenetic Analysis

Phylogenetic analysis was performed via http://www.phylogeny.fr [16], [17]. The sequences were aligned using Muscle v. 3.7 with default settings. The positions with gaps were removed and the curated sequences were used for building Maximum likelihood phylogenetic trees using phyML with default settings, including the WAG substitution matrix. The phylogenetic trees were statistically supported by approximate likelihood-ratio tests using default settings and values between 0 and 1 were obtained, alike bootstrap values. Only approximate likelihood-ratio test values below 0.70 are reported in the trees. Sequences with obvious and large mistakes, be it annotation mistakes or pseudogenes, were not included in the trees, but still listed in the Table S1. For clarification, cosmetic rearrangement of the trees was made using Adobe Illustrator (Adobe, USA).

## Results and Discussion

### Green and Red Algae: A Taxonomic Overview

The red algae consist of up to 10,000 mostly marine species constituting two subphyla, the Cyanidiophytina (including the *C. merolae*, Matsuzaki et al. [18] and *G. sulphuraria*, Barbier et al. [19]) and the Rhodophytinia [20]. The green algae encompass approximately 6,000 species delineated into three major groups [5], These include: (1) the early diverging Chlorophyta or the Prasinophyceae, a primitive group of mostly scale-covered or naked unicells primarily found in marine ecosystems (including *O. tauri, O. lucimarinus* and *Micromonas* sp. [21]–[23]). This group represents the basal stock of green algae; (2) the core Chlorophyta consisting of the Chlorodendrophyceae, a small group of unicellular forms with cell walls comprised of fused scales, the Ulvophyceae (green seaweed/siphons), the Chlorophyceae (including the model organisms, *C. reinhardtii* and *V. carteri f. nagariensis* [24], [25]) and the Trebouxiophyceae, a unique group of terrestrial and lichen algae [26]; (3) The Streptophyta, or the Charophycean Green Algae (CGA). This group emerged between 725 million and 1.2 billion years ago and it is believed that between 450–500 million years ago, an ancestral form of the CGA emerged onto land and ultimately gave rise to land plants. Indeed, much similarity exists between modern land plants and CGA [8], including chloroplast structure and pigmentation, flagellar apparatus substructure, the production of starch reserves and most recently discovered, cell wall biochemistry [27], [28].

The extracellular coverings and especially the cell walls of green and red algae display great structural and biochemical diversity [29]. For example, while the CGA have cell walls similar to land plant cell walls, the walls of Volvocalean clade of the Chlorophyceae are not polysaccharide based but are comprised of an assemblage of glycoproteins. The well-known and –studied flagellate, *C. reinhardtii* has a cell wall containing glycoproteins with homology across the Chlorophyceae. Its structural proteins display a set of glycans that are more diverse and, in some cases, more elaborate than those of the extensins, the equivalent cell wall proteins of terrestrial plants [30]. The glycosylation motifs that govern extensin-type glycosylation according to the contiguous hydroxyproline hypothesis are characterized by the SOOO (where O represents hydroxyproline) sequence usually occurring several times. Showalter et al. [31] used SPPPSPPP to define the class of extensins in their bioinformatic classification of hydroxyproline-rich glycoproteins or HPRGs. SPPP-sequences are ubiquitous in Viridiplantae sequenced genomes and go all the way back to the Rhodophyta. But whether the residues outside the SPPP regions are orthologous in Viridiplantae, is difficult to establish, as there is little sequence similarity between the different SPPP containing proteins. Implications are that the SPPP motif have been recruited several times during evolution and incorporated into different non-orthologous genes or that there can be significant genetic drift in the non-SPPP regions masking orthology.

Beside extensins, mannan and cellulose are also shared between Chlorophyta and CGA cell walls [32]. Mannan is made of ß-1,4 linked mannose and is alike in both Chlorophyta and CGA. Cellulose consists of ß-1,4 linked glucose organized in semicrystalline fibrils. Cellulose has prokaryotic origin and is produced by both fungi, animals and it exists in both the Chlorophyta and CGA, but the molecular organization of the glucan chains into crystalline microfibrils is different. Cellulose is synthesized at the plasma membrane either by linear, terminal complexes as in the bacterium, *Acetobacter xylinum,* or by complexes organized as rosettes. The former produce ribbon-shaped, highly crystaline cellulose microfibrils while the rosettes produce fibrils of circular cross section, lower degree of crystalinity and higher degree of polymerization (see Saxena and Brown [33] for an overview). Both the CGA and terrestrial plants feature the rosette-type complexes and produce cellulose embedded in complex matrix polysaccharides [34]. Linear terminal complexes are expected in the Chlorophyta [35], that in turn, produce cellulose that is not coated with soluble polysaccharides. Surprisingly, it has recently been observed that the genome of the lycophyte, *S. moellendorffii,* possesses genes encoding both rosette-type cellulose synthases and linear bacterial-like cellulose synthases [13].

The cell wall of red algae included in this study, *C. merolae* and *G. sulphuraria* has not been analyzed in detail. However, it is known that another extremophile species from the Cyanidiophytina, *Cyanidium caldarium*, contain cellulose albeit in relatively small amounts, polymers containing galactose, mannose, xylose and glucose and a high quantity of protein (approximately 50%; [36]. The cell wall glycoproteins may be cross-linked via certain tyrosine residues [37], [38]. Genes encoding class III peroxidases have been identified in *Galdieria* [39] that might be involved in crosslinking of these tyrosines. Weber and colleagues have compared the genome of *G. sulphuraria* to that *C. merola;* two closely related taxa of which the latter is considered wall-less. They observed that *G. sulphuraria* has genes encoding putative fucosyl- and galactosyltransferases that the wall-less family member lacks [19].

#### Classification system

The complete proteomes of species *C. reinhardtii*, *V. carteri f. nagariensis*, *C. merolae* and *G. sulphuraria* were screened for putative GTs by utilizing the whole CAZy database as bait, and not just plant specific CAZy members. This approach allowed for possible orthologies, e.g., prokaryotic, fungal or mammalian genes, to be identified. In *Physcomitella patens,* a number of genes orthologous to sequences of fungal origin were found, and similar results would go unnoticed unless the whole CAZy database was used [13]. *Micromonas* sp., *O. tauri* and *O. lucimarinus* were already annotated in the CAZy database and they were included as is, but in cases of ambiguity manual BLAST has been used. A conservative approach on identifications of GT’s has been chosen to avoid identifications of false positives. Mis-annotations tend to be hard to remove from public databases and later annotations of orthologous genes could be based on false positives from present work.

There are two possible sources of false negatives (genes that encode GTs but not caught by the screen): Firstly, sequences that are too divergent to be recognized by our method of comparing to already classified GTs; and, secondly, GT-encoding gene models that did not appear to be solid enough to ever make from ‘all models’ to ‘filtered’ or ‘best models’ as defined by the curators of the genome projects. The latter possibility was investigated using *Chlamydomonas* as an example. Its all model proteome file is much larger than the filtered models proteome – it comprises 24191 gene models that have no equivalents in ‘best models’. These were searched for putative GTs, and none were found. This lead us to conclude that false negatives are comprised entirely of sequences (from all or filtered models) too divergent to be recognized as GTs by state of the art bioinformatics methods.

With this manuscript, a naming convention on green and red algal GTs is introduced. The naming convention follows that already introduced in plants, which originates from Joint Genome Institute instructions on naming of de novo sequenced genomes. A GT that is clearly orthologous to a known and named GT from another species will share that name, to be preceded by species initials. When there is no clear orthology, no known (putative) function or no pre-established naming convention, then a gene is named GT<CAZy-family><clade letter><number>. The clade letters are assigned based on the phylogenetic trees and existing naming. Table S1 lists gene names for all families and all species.

The principle of naming genes after their orthologs based solely on phylogenetic analysis is not without pitfalls. For example, clades with one characterized activity may well contain additional activities, but are named after the first discovered activity. So although a conservative approach to determining orthologies is adopted, there are probably genes that shall have to be renamed as additional biochemical evidence becomes available.

**Figure 3:**
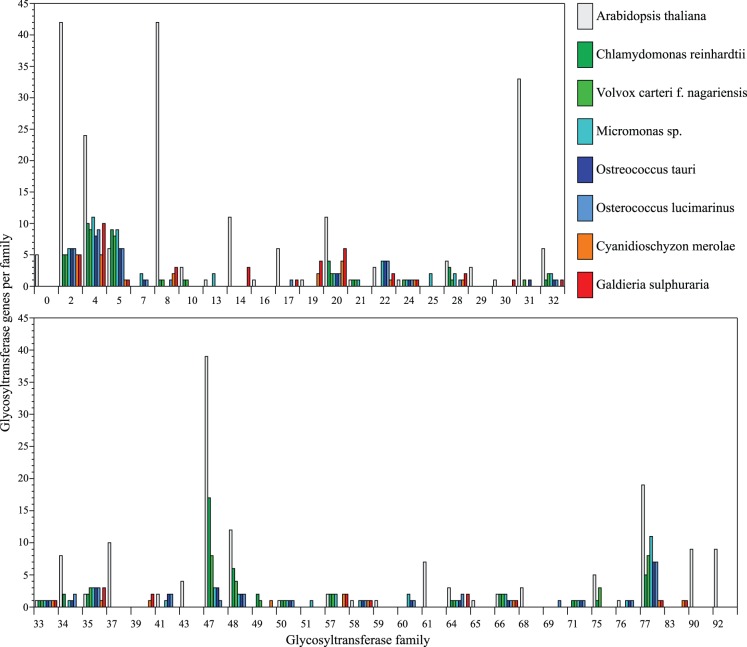
The distribution of glycosyltransferases in analyzed genomes. Arabidopsis is included as reference genome.

The general overview of GTs found in the green algal genomes is presented in Figure 3. The number of GTs found in the green and red algal species are generally lower, both in total and relative to number of genes present in the genomes, then what is observed in primitive and higher plants (Table 1; [13]). Three particular aspects of the physiology of embryophytes increase the number of GTs in their genomes: (1) Cell wall biosynthesis as the more complex cell walls of embryophytes requires a larger number of GTs for biosynthesis; (2) a richer set of protein glycosylation; and (3) glycosylation of secondary metabolites, where a large proportion of GT1 is involved in secondary metabolite biosynthesis. Xyloglucan, (glucurono- arabino-)xylan and pectin are only found in Streptophyta so the GTs involved in their biosynthesis are not found in green or red algae. The large majority or all of GT2, GT8, GT34, GT37, GT43, GT47 and GT77 are consequently missing in green and red algae. A richer set of GTs is also observed in GT14 and GT31. No plant activity is known from GT14 but the close homology to DUF266, which contains a GT putatively involved in AGP biosynthesis, suggests that the plant sequences in GT14 are also involved in AGP biosynthesis [40], [41]. GT31 contains GTs involved in N-glycosylation but the majority of the GTs are generally believed to be involved in AGP biosynthesis [42].

**Table 1:** Number of GTs found in the different organisms analyzed.

Species	Total number of GT	Percent of genome being GTs
*Arabidopsis thaliana*	462	1.81^1^
*Selaginella moellendorffii*	288	1.45
*Physcomitrella patens*	370	0.80
*Chlamydomonas reinhardtii*	76	0.50
*Volvox carteri f. nagariensis*	67	0.43
*Micromonas sp.*	77	0.77
*Ostreococcus tauri*	52	0.66
*Ostreococcus lucimarinus*	57	0.75
*Cyanidioschyzon merolae*	32	0.64
*Galderia sulphuraria*	54	0.75

1 The number of arabidopsis GTs include the 122 members of GT1, but do not include the 99 GTs not assigned to a CAZy family.

The GT1 comprises 121 sequences making it the largest family in arabidopsis. None of the red or green algae have more than a few GT1 and none of these are UGTs involved in small molecule glycosylation, possibly reflecting the adaption that embryophytes have made in order to be able to survive the harsher terrestrial environment and evolve associated complex reproductive systems using for example glycosylated volatile compounds as pollinator attractants.

The next two sections present: (1) some of the different GT families found in the green and red algae, providing new insight into the comparative physiology of specific taxa; and (2) a section on the biosynthesis of specific polymers, for example, protein glycosylation and GPI anchor biosynthesis.

### GT2

The CAZy GT2 family is very large, including many enzymes not found in plants, such as chitin synthase and hyaluronan synthase. Plant, green algal and red algal related activities in GT2 are involved in protein N-glycosylation (see later paragraph on protein glycosylation) and cell wall biosynthesis. In plants, GT2 is one of the major cell wall-related GT families with backbone synthases for all but xylan and pectin synthesis [43], [44].

The cell wall-related GTs from GT2 all belong to the CESA superfamily. This superfamily can then be divided into three subfamilies: a) a CESA subfamily with CESA, CslB, and CslD through to CslJ; b) a CslA and CslC subfamily; and c) a subfamily of linear, terminal complex- forming cellulose synthases. The CESA superfamily has been the focus of much interest. Phylogenic comparisons between the CESAs of the Viridiplantae and the recognition of the absence of Rhodophyta-based CESA superfamiliy members have been previously reported [45]. Additionally, red algal linear complex-forming CESAs have been identified previously. However, in *C. merolae* and *G. sulphuraria,* no linear complex-forming CESAs could be found; an indication that only part of the red algal lineage has retained these complexes [46]. The same observation could be made in the Chlorophyceae genomes. Linear complex-forming CESAs have been described in ultrastructural studies of the Chlorophyceae, but could not be found in their previously-analyzed genomes (Tsekos 1999). CGA, bryophytes and lycophytes have retained their linear complex-forming CESA (Harholt et al. 2012), a characteristic lost in gymnosperms and angiosperms. The rosette-forming CESAs typically found in gymnosperms and angiosperms are of apparent CGA origin as none have been discovered outside Embryophyta and Charophyta [47].

The green algal specific clade orthologous to CslA and CslC with unknown function is named CslK as a new distinct clade in the CESA superfamily as no clear orthology to either CslA or CslC can be established (Figure S1). The CslK was also reported by Yin et al. [45] but not named.

### GT4

The GT4 family is large, diverse and contains many different activities. In order to accommodate this feature, the family is split into smaller groups, based on shared activity and phylogeny.

In multicellular land plants, the disaccharide, sucrose, is mainly synthesized in “source” tissues by sucrose phosphate synthase and then catabolized via sucrose synthase in the “sink” tissues. Sucrose phosphate synthase and sucrose synthase have been thought to be found only in land plants as neither had previously been found in green or red algal genomes. Sucrose phosphate synthase and sucrose synthase are both of cyanobacterial origin so it might be expected that they should also be present in green algae, the ancestors of the land plants [48]. However, attempts to measure sucrose phosphate synthase activity in *C. reinhardtii* have been unsuccessful and this alga does not accumulate sucrose during photosynthetic activity [49]. This correlates well with our findings where we also noted the lack of sucrose phosphate synthase in our select green algal taxa. *Chlamydomonas* and the green algae studied here are all unicellular, i.e., of low morphological complexity. However, in the multicellular green seaweed, *Ulva australis.* sucrose phosphate synthase has been identified (Hawker and Smith 1984). It might be argued on these limited observations that the presence of this enzyme might be associated with more complex morphology, in this case, a multicellular thallus. Yet further complicating the present situation, the unicellular *Chlorella* sp. has been reported to have sucrose phosphate synthase activity [50]. While a much more thorough survey of sucrose synthesizing enzymes in the green algae is required, we might speculate that unique, taxon-specific changes in the physiology of the analyzed green algae are reflected by the loss of sucrose phosphate synthase and sucrose synthase from their genomes. With respect to red algae, sucrose is not produced as soluble C storage and hence the lack of sucrose phosphate synthase and sucrose synthase genes is expected [51].

Two subclades of GT4 with physiology related activities are the SQD2 and DGD clades involved in chloroplast membrane development (Figures S2 and S3). All of the analyzed genomes of this study have SQD2 orthologs where as only Chlorophyte algae have DGD orthologs.

GT4 furthermore contains sequences involved in GPI anchor biosynthesis (PIGA; Figure S4), N-linked protein glycosylation (ALG2 and ALG11; Figure S5 in the Supporting Informaiton; both are discussed below) and some clades with unknown activity (Figure S5). The clades with unknown activities contain a mixed presence of the analyzed genomes.

### GT5

The starch synthases, both soluble and granular bound forms, are conserved among all the Viridiplantae analyzed (Figure S6). Arabidopsis contains four soluble starch synthases and one granular bound starch synthase and the green algae have orthologous sequences to all of these five. The red algae do not produce starch of similar structure as Viridiplantae, but produce floridean starch [51]. This is also reflected in GT5 as the orthologous starch synthases found in red algae are quite divergent from the Viridiplantae starch synthases.

### GT8

GT8 is a somewhat large and divergent family, containing several different plant activities. The GAUT and GATL subfamily contains cell wall related activities, with GAUT1 being a homogalacturonan synthase and others being putatively involved in homogalacturonan or xylan biosynthesis [52]–[55]. The non-cell wall-related sequences are involved in galactinol synthesis [56]. The PGSIP subfamily was initially reported to be involved in starch synthesis, but recently part of this family (arabidopsis PGSIP1/GUX1 and PGSIP3/GUX2) was demonstrated to be involved in xylan side chain decoration [57]–[59]. The PGSIP subfamily is divergent and contains three clades, containing PGSIP1-5, PGSIP6, PGSIP7 and 8, respectively [60]. The only GT8’s found in the present study are orthologous to the PGSIP6, PGSIP7 and PGSIP8 clades, respectively (Figure S7). Both green and red algae have PGSIP6 orthologs whereas only the red algae have PGSIP7 and 8 orthologs. No activity has been reported for these clades so clear interpretations of our findings are difficult.

### GT17

Only an *O. lucimarinus* sequence is present in CAZy and two fragments from have been reported for any plant GT17 member and no functionality can be proposed for the *O. lucimarinus* GT17 at this time.

### GT20

The GT20 family consists of trehalose synthases with characterized members from both pro- and eukaryotes, as listed in CAZy. All the genomes analyzed contain GT20 orthologs, suggesting that the functionality of trehalose as an osmo-regulator has been conserved throughout the Viridiplantae (Figure S8).

### GT39

Animal, fungal and bacterial members of GT39 are involved in mannosylation of serine or threonine via *O*-linked glycosylation [61]. This activity has not yet been reported in the Viridiplantae. Two *G. sulphuraria* and one *C. merolae* orthologous sequences were identified in this study. Orthology could be established based on similarities in both topology and sequence (results not shown). The activity of these GTs is not known and it is an open question whether the red algae have retained them or acquired them via horizontal gene transfer.

### GT48

All plant members of GT48 are expected to be β-1,3-glucan synthases based on orthology to proven β-1,3-glucan synthases and complementation studies [62], [63]. The Chlorohyte family GT48 sequences found in this study are also probable β-1,3-glucan synthases (Figure S9). GT48 members were not identified in our prasinophyte or red algal genomes. In silico transmembrane helix predictions of the green algal sequences identified all envisage the same domain structure as observed in plants and fungi. In addition to the transmembrane helix structure similarities between plant and green algal GT48 members, the intron exon structure compartmentalized into two distinct groups; one group with few introns and a second group highly fragmented with many introns, with both groups conserved in Viridiplanteae [64]–[67]. Upon aligning known β-1,3-glucan synthases from fungi and plants with Chlorophycean sequences, a highly conserved domain spanning part of the catalytic loop and the C-terminus of the protein is easily recognized where as the N-terminal region is specific for either Chlorophycean, fungal, or plant sequences (results not shown). In the *C. reinhardtii* zygote cell wall and during vegetative growth, the presence of callose has been demonstrated by aniline blue staining and susceptibility to 1,3-glucanase degradation [68]. Callose in land plants is not a major cell wall component but is involved in for example defense, pollen tube development and in plasmodesmata formation. As only part of the catalytic domain and the C-terminus of GT48 is conserved between green algal and plant ß-1,3-glucan synthase and as this domain is orthologous to fungal GT48 sequences, we cannot infer a physiological function in Chlorophyceae from that in plants.

The red algae included in this study do not posses orthologous GT48 sequences, but as observed in green algae with only *C. reinhardtii* and *V. carteri f. nagariensis* possessing GT48, it cannot be conclusively shown that the red algae are lacking GT48 members in general.

### GT51

The cyanobacterial derived peptidoglycan layer sandwiched between two membranes in chloroplasts plays a particular role in algal evolution. It is widely accepted that the chloroplast of modern day photosynthetic eukaryotes was derived from endocytosis/endosymbiosis of a bacterium, most likely a cyanobacterium. Green algae, red algae and glaucophytes are modern derivatives of this simple, primary endosymbiosis and all other algae have plastids derived from endosymbiosis of green and red algae. In plastids evolved from primary endosymbiosis, the peptidoglycan-rich wall would be predicted to be located between the two membranes of the chloroplast and indeed, in modern day glaucophytes, a peptidoglycan component is found here [69]. However, no such structure has yet to be identified in green or red algae. In the green algae, only one taxon analyzed, *Micromonas* sp. *RCC299,* has a gene encoding a GT51. Members of this family, the murein polymerases, catalyze the last step in the synthesis of peptidoglycan biosynthesis. Murein polymerases may comprise a penicillin-binding sensitive transpeptidase domain in addition to the transglycosylase domain that assigns them to family GT51. Plastid division inherits mechanisms and gene-sets from the cyanobacterial ancestors of the chloroplasts. Interestingly, recent work has shown that the chloroplasts of *P. patens* [70] and *Selaginella nipponica* [71] but not flowering plants are sensitive to peptidoglycan synthesis-affecting antibiotics. Genes coding for the synthesis of peptidoglycan, including GT51, are found in *P. patens and S. moellendorffii* [13], [72]. Both *C. reinhardtii* and arabidopsis lack murA-D and F that catalyze earlier steps in murein synthesis while *P. patens* and *S. moellendorffii* have them [13], [73]. Curiously, *Micromonas* sp. RCC299 lacks murA-D and F while *Micromonas pusilla* has them (data not shown). This observation suggests that the peptidoglycan biosynthetic machinery conserved in *S. moellendorffii* can be traced back to early algal ancestors, but its value as a high-level taxonomic discriminant may be questioned if conservation of the biosynthetic pathway varies within a single algal genus.

### GT83

The finding of GT83 members in Rhodophyta is an interesting discovery. The only identification of GT83 sequences in the Viridiplantae to our knowledge is in *P. patens* [13]. GT83 consists of bacterial sequences that encode enzymes involved in lipopolysaccharide biosynthesis. Even though homology exists between the Rhodophyte GT83s and the *P. patens* GT83, horizontal gene transfer is the most likely explanation for the occurrence of GT83 in such evolutionary diverse species.

### GT Families with no Entries from Plants

Members of GT49 are involved in synthesis of poly-N-acetyllactosamine in animals. Poly-N-acetyllactosamine is a polymer consisting of disaccharide repeats of N-acetylglucosamine and galactose. Beside GT49, GT7 is involved in its biosynthesis and in humans, complex interactions between GT49 and GT7 proteins have been identified [74]. GT49 members were also found in Chlorophyceae and the red alga, *C. merolae.* Since neither of these have GT7 members it is not clear what activity the GT49 members have.

GT25 contains *Micromonas* sp. sequences but not *Ostreococcus* sp., GT60 also contain only prasinophyte sequences, and here distant orthology can be observed with a brown alga (*Ectocarpus siliculosus (Dillwyn) Lyngbye*) and *Dictostelium*. In *Dictostelium* the function has been identified as a mucin type *O-*glycosylation or more specifically a GalNAC transferase that has Hyp as acceptor. But as the similarity is low between the GT60 sequences, trying to infer function is speculative at best.

### Protein N-glycosylation

#### N-glycosylation

The N-glycosylation of proteins is highly conserved in all eukaryotes and follows the general scheme of glycan biosynthesis upon a lipid anchor, transfer to target protein, trimming by hydrolases and then possibly, additional glycosylation steps. This process starts in the ER and only the biosynthesis of the complex N-glycans occur in the Golgi apparatus. N-glycan biosynthesis has recently been reviewed [75]–[77]. The GTs involved in N-glycan biosynthesis have been elucidated and orthologous plant sequences exist. So, it is hypothesized that plant N-glycan biosynthesis to a large degree, occurs as it is believed to occur in animals and fungi. Only in the formation of complex N-glycans do plants deviate. Plants possess additional activities that synthesise a complex N-linked glycan containing a trisaccharide structure known as Le^a^ and lack activities involved in ß-1,4-galactosylation, sialylation and additional branching as in animals and fungi. The genes encoding the plant specific activities are known and described [78].

The biosynthesis of N-glycans has been well-studied with the first steps being elucidated in the 1960s. Surprisingly, the biosynthesis involves production of a lipid-linked oligosaccharide precursor structure. This structure is then transferred en bloc to the target protein. N-glycan addition then occurs on asparagines in the sequence context Asn-X-Ser/Thr. To date, all eukaryotic cells analyzed produce N-glycans and the protein involved in the earliest biosynthetic steps making the dolichol-oligosaccharide precursor as well as several subsequent processing reactions in the ER are highly conserved in the species analyzed to date.

**Figure 4:**
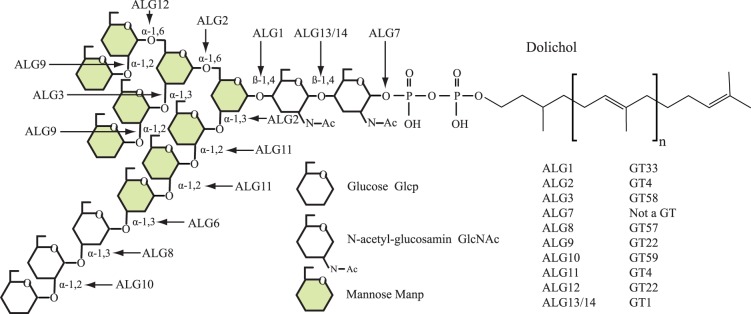
Schematic illustration of the N-linked glycan before its transfer onto the protein. The linkages and name of biosynthetic enzyme is presented in the figure along with an overview of CAZy family for the different glycosyltransferases.

The glycosyltransferases involved in the biosynthesis of the ER-localized core glycan is found in GT1, GT2, GT4, GT22, GT33, GT57, GT58 and GT59. The initial cytoplasm-based addition of GlcNAc and mannose to the inositol anchor is conserved in both red and green algae (ALG13, ALG14 both GT1, ALG1/GT33 (Figure S10), ALG2/GT4 (Figure S11) and ALG11/GT4 (Figures. S11 and 4). After the inversion of the glycan structure from the cytoplasmic side to the lumen of the ER, the subsequent mannosyltransferases use dolichol-P-mannose as sugar donor. The activity transferring mannose from GDP-mannose to dolichol is DPM1 from GT2 (Figure S12). DPM1 is found in all green and red algal genomes analyzed. The dolichol-P-mannose is used for additional mannosylation by ALG3 from GT58 (Figure S13), ALG9 and ALG12 from GT22 (Figure S14; Figure 4). None of these activites are found in the Chlorophyceae. DPM1 is transcribed, as ESTs can be found (BLAST against NCBI EST database, results not shown) indicating activity is present. DPM1 also supplies substrate for the mannosyltransferases involved in GPI anchor biosynthesis, possibly explaining its occurrence and transcription in Chlorophyceae [79]. Biochemical characterization of N-linked glycans in *V. carteri f. nagariensis* showed that the highest observed mannosylation was with five mannoses [80]. The Chlorophyceae phenotype appears to be due to complete lack of ER luminal mannosylation.

The mannose decorations are further glucosylated by ALG6 and ALG8 of GT57 (Figure S15), and ALG10 of family GT59 (Figure S16) using dolichol-P-glucose as substrate (Figure 4). The dolichol-P-glucose is provided by ALG5 from GT2, orthologs are found in all genomes analyzed but prasinophytes (Figure S12). The initial glucosylation is catalyzed by ALG6 and it too has orthologs in all genomes analyzed except those of the prasinophytes. Biochemical activity has also been shown in *V. carteri f. nagariensis* using Dol-PP-(GlcNAc)_2_-(Mannose)_5_ as substrate and dolichol-P-glucose as substrate [80]. Biosynthesis continues with additional glucosylation by ALG8 orthologs, which again is found in all genomes analyzed but prasinophytes. The last glucosylation by ALG10 orthologs is not found in any green and red algal genomes analyzed. The above findings are in agreement with Gomord et al. [76].

The glucosylation is thought to be needed for proper transfer of the glycan from the dolichol anchor to the target protein as yeast knock outs of ALG6/8/10 show improper transfer [81]. Prasinophyceaen and Chlorophyceaen species utilize N-linked glycoprotein, for example, in flagella. Hence, the missing glucosylation is apparently not a problem for correct transfer to target protein in these species [82], [83].

The later addition of sugar decorations in the Golgi apparatus is not fully conserved among animals, fungi and plants. Some features such as addition of α-1,3 fucose to the innermost GlcNAc is common among plants and invertebrates whereas decoration of the mannoses is dissimilar between and plants and other eukaryotes, see Bardor et al. [84] for an introduction. Two types of N-linked glycans are found in plants. The high mannose type, which is not modified in the Golgi and a complex N-linked glycan that is unlike the complex N-linked glycan found in vertebrates. Based on enzymatic degradation and lectin binding studies, both high mannose and complex N-linked glycans have been reported from *Tetraselmis striata* from Volvocales [85] and biochemical analysis of N-linked glycoprotein from *V. carteri f. nagariensis* shows the occurrence of xylosylated N-linked glycans [86]. Based on the findings in the present study, complex N-linked glycans, as observed in plants, are not present in either green or red algae. The core β-1,3 fucosylation of the innermost GlcNAc is apparently present in the Chlorophyceae as exemplified in *V. carteri f. nagariensis* and *C. reinhardtii* orthologs of the FuctA activities from arabidopsis (found in GT10); [87], [88]. Orthologous sequences responsible for the remaining Golgi localized biosynthesis of N-glycans in green or red algae are not found in any of the genomes analyzed (GT13, GT16, GT61, GT31 and GT10 (α-1,4-fucosyltransferase); [89]. One GT10 from *G. sulphuraria* as reported by Barbier et al. [19] and *Micromonas* sp. along with two GT10s from *O. tauri* and *O. lucimarinus* were also found. But the sequence identity is low and if other non-embryophytes are included, e.g. a GT10 from the pelagophyte *Aureococcus anophagefferens* it is more related to the Chlorophyceae and plant GT10s than to the *G. sulphuraria* and prasinophyte sequences (results not shown). This raises the question whether functional orthology is conserved.

Xylosylated N-linked glycans have been reported in *V. carteri f. nagariensis* but the GT61 activity adding the xylosyl residue in arabidopsis appears to be missing [86], [90].

### O-Glycosylation

*O-*glycosylation exists in fungi, animals and plants. However, as opposed to N-glycosylation, *O-*glycosylation is not as conserved between the three kingdoms. In Viridiplantae, two unique classes of proteins are *O-*glycosylated; extensins and arabinogalactan proteins (AGP). Both classes are involved in cell wall functionality [91], [92].

Certain *O-*glycosylations are highly conserved from bacteria to mammals. The GlcNAc transferase activity of GTs found in GT41 catalyzes the transfer of GlcNAc onto Ser or Thr and is conserved in all kingdoms except Archaea. Two groups of sequences cluster in GT41, a SPY related and SEC related group (plant names are used as plants contain orthologs in both groups) [93]. Not all organisms contain both groups; bacteria for example only contain SPY homologs. In the Viridiplantae, all known embryophyte genomes analyzed contain both groups, whereas analyzed green algal genomes only contain SPY homologs. In Olszewski et al. [93] the red alga, *G. sulphuraria,* was reported to contain both SPY and SEC orthologs, which was confirmed.

Protein *O-*mannosylation is believed to be restricted to animals and fungi but is now also evident in prokaryotes [94]. However, we report the finding of orthologous sequences of the primary activity, found in GT39, responsible for the *O-*linked mannosylation of serine or threonine [94]. Two *G. sulphuraria* and one *C. merolae* orthologous sequences were identified. Based on agreement in transmembrane helix predictions, location of conserved arginine residues involved in complex formation, conservation of aspartate and glutamate residues and approximately 30% pairwise identity between fungal, human and red algal sequences, orthology could be established (results not shown). The presence of mannosyl transferase activity in red algae will require further biochemical characterization.

The *O-*linked mannose may be further glycosylated with a variety of motifs. Some of the genes involved in these glycosylations are known. One family, namely GT71, is involved in biosynthesis of a *O-*linked pentameric mannan. Chlorophyte GT71 members can be found but since the GT39 and GT15 activities required for acceptor biosynthesis were only found in red algae (see above) or not found at all, respectively, we cannot infer function of the green algal GT71s.

In GT7, only sequences from prasinophytes were found and upon blasting against NCBI protein database scores around 10^−20^ were obtained against animal β-1,4-galactosyltransferases. These proteins are involved in protein glycosylation and lack of this activity leads to a range of disorders in humans [95]. No relation can be observed with the activity found in the prasinophyte sequences and the animal activity. Therefore, a unique prasinophyte activity could therefore be anticipated.

The Viridiplantae have unique *O-*glycosylation patterns leading to two distinct classes of *O-*glycosylated proteins: Arabinogalactan proteins (AGP), and extensins. Both groups belong to a group of proteins known as hydroxyproline-rich proteins. AGP and extensin are glycosylated as results of three modifications, a hydroxylation of proline and then glycosylation of some of these hydroxyprolines (Hyp) and additionally galactosylation of some serines.

**Figure 5:**
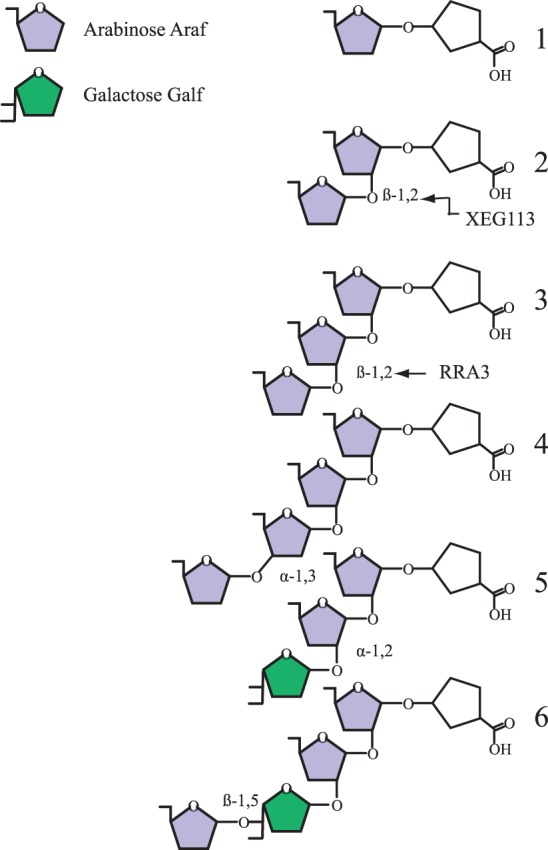
Illustration of the most common Hyp arabinosides so far found in Chlorophytes, Charyophytes and plants. Structure 1–3 is shared among viridiplantae (Lamport and Miller, 1971, Harholt, Petersen and Ulvskov, unpublished). Structure 4 has so far only been observed in Charophycean green algae and plants (Lamport and Miller, 1971, Harholt, Petersen and Ulvskov, unpublished). Structure 5 and 6 is found in at least *C. reinhardtii* but not been reported in plants [101]. Structure 5 and 6 can be methylated at either C-6 of the galactose or C-3 at the ultimate arabinose [101].

Extensins are structural wall proteins glycosylated on contigous Hyp residues [96]. Glycosylation in land plants comprise single α-1,3 galactosyl residues onto serine and short arabinosides attached to Hyp-residues often arranged in SOOO motifs [42]. The arabinosides are unusual in that the three innermost arabinosyl residues are linked β-1,2. The fourth arabinosyl residue is α-1,3- while the configuration of the low-abundance fifth residue is unknown [97] (Figure 5). Extensin-like epitopes have been identified using monoclonal antibodies in the late divergent CGA [98] including the Zygnematalean taxon, *Cosmarium reniforme* [99] and our mass spectrometric analysis of the cell walls of *Penium margaritaceum* also belonging to Zygnemetales suggests extensin arabinosylation that cannot be distinguished from that of land plants (Harholt, Petersen, Ulvskov, unpublished). Green algal Hyp-glycosylation has been studied most extensively in *C. reinhardtii*, which demonstrate more elaborate glycans with a richer variation in sugars and linkages [100]–[102] (Figure 5). A common core of the two β-1,2 linked arabinofuranosyl residues closest to the Hyp is, however, conserved in all extensin-like structures examined so far (Figure 5). Additionally is the structure containing three arabinoses found in both Chlorophytes and GCA (Lamport and Miller 1971, Harholt, Petersen, Ulvskov, unpublished; Figure 5).

None of the β-arabinosyltransferases involved in extensin biosynthesis have been unambiguously identified. Putative extensin β-arabinosyltransferases have, based on mutant phenotypes, been identified. Egelund et al. [103] suggested that the RRA proteins are involved in extensin arabinosylation. Gille et al. [104] presented evidence that Xeg113 was involved in the third arabinosylation of extensin. This was further supported by thorough biochemical analysis of knock out mutants of respective genes and the putative function on adding the second and third arabinose was confirmed [105].

**Figure 6:**
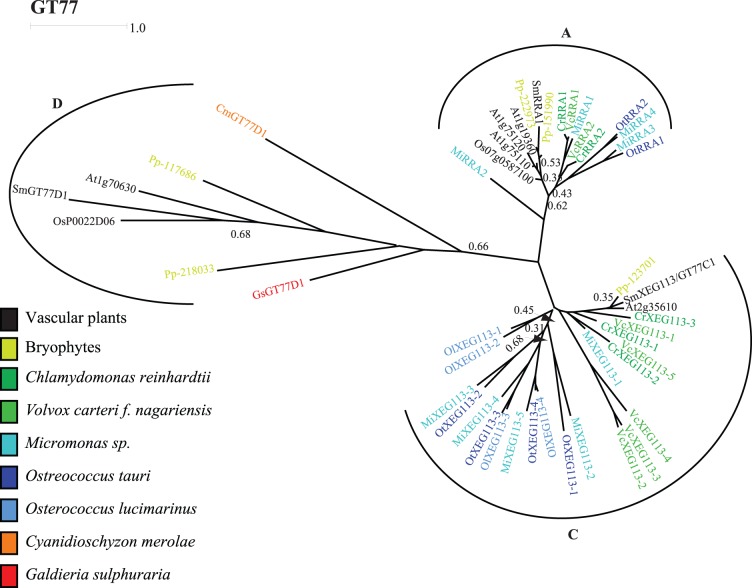
CAZy family GT77. Characterized members of GT77 are involved in extensin biosynthesis as arabinosyltransferases (A- and C-clades) and rhamnoglacturonan II biosynthesis as xylosyltransferases (B-clade. Not shown – no algal members). Chlorophyte orthologs to the extensin arabinosyltransferases RRA and XEG113 from arabidopsis could be identified (At1g75120: AtRRA1, At1g75110: AtRRA2, At1g19360: AtRRA3 and At2g35610: XEG113). Additionally, rhodophyte members could be identified belonging to the GT77D clade with unknown activity. Rice XEG113 orthologs have been identified, but was omitted from the tree due to apparent annotation mistakes in the protein sequence. The scale bar indicates the average number of amino acid substitutions per site.

RRA and XEG113 are members of GT77 residing in GT77A and GT77C, respectively and both clades comprise sequences from all analyzed algal species (Figure 6). All C-clade members are named XEG113 due to sequence identity of more then 30% within the conserved regions of the proteins. Orthology comprising all species is not guaranteed, however. Missing GT75-sequences in some taxa raise doubt about the function of XEG113 and RRAs in these instances. UDP-Ara*p* is the naturally occurring nucleotide sugar of arabinose so arabinosyltransferases probably require the participation of a GT75 mutase that catalyzes the UDP-ara*p*<->UDP-ara*f* interconversion [106]. The finding of GT75s in the genomes *C. reinhardtii* and *V. carteri f. nagariensis* is thus not surprising (Figure S17) but its absence from prasinophytes is somewhat confusing. The prasinophytes included in this study are considered wall-less but we have recently argued that this characteristic deserves a closer study [107].

The glycosyltransferases involved in transferring the first, fourth, and fifth arabinose have so far not been identified. In GT47, the E clade only contains plant and green algal sequences (Figure S18 in the Supporting Information). As GT47 contains inverting activities, this clade could be responsible for the α-1,3-arabinosyltransferase activity that adds the fourth arabinosyl to extensin arabinosides. The arabinosyltransferase activity needed for adding the innermost arabinose and fifth arabinose are not known. Good candidate clades could not be identified in this study, suggesting that the activity could be found outside CAZy. The arabidopsis serine galactosyltransferase activity needed for the galactosylation of the Ser has been submitted to GenBank but not yet published (Accession: BAL63044.1; Saito F, Suyama A, Oka T, Yoko-o T, Matsuoka K, Jigami Y. and Shimma Y unpublished). Orthologs were found in all the analyzed Chlorophyte species but not in Rhodophytes (results not shown).

If the Chlorophyceae specific glycan structures are synthesized by GTs unique for the Chlorophyceaea then these GTs will most likely not appear in this study due to the comparative approach used. In plants several new classes of GTs have been identified that were unrelated to known GTs and this might be the case in algae as well [108]–[110].

Very little is known on AGP glycan structure biosynthesis but is generally assumed that the plant members of GT31 comprise activities involved in adding galactosyl units to *O*-glycans of AGPs [111]. AGPs are cell wall glycoproteins that can be attached to the plasma membrane through GPI anchors, reviewed recently by Seifert and Roberts [112] and Qu et al. [113]. Only fragments of a *V. carteri f. nagariensis* sequence orthologous to GT31B clade members and a fragment of a *C. reinhardtii* sequence with low sequence orthology to GT31 can be identified. The *C. reinhardtii* sequence was found in an initial draft genome and not in the final v4 proteome. A *Cryptococcus* GT31 is also annotated in the CAZy database, but this sequence does not posses any close similarity to plant, *V. carteri f. nagariensis* or *C. reinhardtii* GT31. In rice a putative GT not classified into CAZy with DUF266 domain have been implicated in AGP biosynthesis [40], but neither of the red or green algae in our analysis contain orthologous sequences (results not shown). So neither of the two putative or confirmed GTs involved in AGP biosynthesis has been found in the Chlorophyte or Rhodophyte genomes.

AGP, or specifically AGP antibody epitopes, have been observed in green algae, which is contradictionary to the lack of genomic evidence for AGP in green algae [114], [115]. But both of the reported findings of AGP are from multicellular algae which could implicate that unicellular or primitive multicellular algae have lost the biosynthetic pathway for AGP biosynthesis The algae studied in the aforementioned papers are members of the core Chlorophyta (Chlorophyceaea and Ulvophyceae), taxa that are separated from the CGA and ultimately the land plants by a time span of approximately 1 billion years.

### GPI-anchor

All GPI-anchors have similar chemical structures, with minor differences between different kingdoms. The core structure of the anchor molecule comprises a sugar moiety and a phosphatidylinositol molecule, linked to two long-chain fatty acids. The sugar moiety is composed of a α-1,6-GlcNAc linked to the phosphatidylinositol and three mannoses α-1,4; α-1,6 and α-1,2 linked to the GlcNAc, respectively [116]. The biosynthetic GTs involved in the core structure biosynthesis is well described and are all named PIG. The GlcNAc transferase of GT4 (PIG A) is present in green algae but not in the red algal genomes analyzed (Figure S4). As this activity is the core activity for GPI anchor biosynthesis, it implies that at least the red algae included in this analysis and possibly the entire assemblage of red algae lack GPI anchors as known from other organisms. The α-1,4 mannosyl transferase of GT50 (PIG-M) is found throughout all Viridiplantae genomes analyzed. But the next mannosyl transferase, the α-1,6 activity of GT76 (PIG-V), is missing in *C. reinhardtii* and *V. carteri f. nagariensis*, but is present in *Ostreococcus* and *Micromonas*. The same goes with the last α-1,2 mannosyltransferase activity. This indicates that either GPI anchors are missing in Chlorophyceae, or that the protein GPI-anchor linkage is mediated via the first mannose, which in certain species can have ethanolamine side groups as reported by McConville et al. [117]. But this would be rather unique as all other kingdoms analyzed so far have the conserved core GlcNAc-(Man)_3_ structure. The function of the glycan structure is not fully understood and truncated glycan structures can mediate proper targeting and recycling in the plasmamembrane, though with decreased diffusion constants [118]. Additional side groups of carbohydrates have been observed in GPI anchors but only one activity (PIG-Z) is known and this activity is missing in the whole Viridiplantaea kingdom [117], [119]. In plants, the GPI anchor is essential for correct cell wall structure. Plants lacking proper GPI anchor due to knock out of PIG-M, a GT involved in GPI anchor glycan biosynthesis, show an embryo lethal phenotype and severe cell wall defects [120]. As for AGP presence, the lack of GPI anchor or only apparent presence of truncated GPI anchors, could be due to the single celled organisms analyzed. In animals, GPI anchored proteins are involved in range of diverse roles such as signal transduction, immune responses and pathogenesis of parasites [121].

## Conclusion

Mapping of the red algal and chlorophyte GT-repertoires resulted in identification of (1) glycosylation processes that are shared among terrestrial plants and either animals or fungi. These include, for example, the core part of protein N-glycosylation and trehalose synthesis. These processes, which are widespread among eukaryotes, provide little phylogenetic and evolutionary insights. (2) No known activity was identified which was only shared between red and green algae and not also with animal or fungi. (3) Additionally, there are processes that comprise the entire green plant lineage but are absent from red algae. The most important example of such general and ancient green plant specific processes include synthesis of Hyp-linked arabinosides. It is noteworthy that while the GTs involved in this process are clearly conserved from prasinophytes to arabidopsis, a similar conservation of proteins that carry these arabinosides cannot be detected. (4) GTs could be observed that were shared between core chlorophytes and embryophytes, GT75 being a prime example. (5) Then there are families of GTs that are represented throughout Viridiplantae but where major changes are observed. The CesA superfamily of GT2 is a significant example as this family faithfully traces evolution and may turn out to be instrumental for classification purposes as more taxa are genome sequenced. (6) Finally, there are families that are present in CGA and Embryophytes but not in Chlorophytes. Members of GT8 involved in homogalacturonan synthesis for example may be seen as a defining feature of CGA and Embryophytes relative to Chlorophytes and is a prime example of the biological, evolutionary and taxonomic significance of cell wall features.

## Supporting Information

Figure S1 **CslA and C of GT2 are involved in mannan and xyloglucan backbone biosynthesis in plants, respectively.** The chlorophyte CslK clade appears ancestral to the plant orthologs and their function is unknown. Some branches are kinked to decrease the space requirements of the figure, the total branch length is still correct. The scale bar indicates the average number of amino acid substitutions per site.
(EPS)

Figure S2 **UDP-sulfoquinovose synthases of GT4, which is found in all the analyzed divisions of Chlorophyte and Rhodophyte algae.** The scale bar indicates the average number of amino acid substitutions per site.
(EPS)

Figure S3 **Digalactosyldiacylglycerol synthases are located in GT4 and is involved in galactolipid biosynthesis in the chloroplast.** All the analyzed chlorophyte algae have orthologs. The scale bar indicates the average number of amino acid substitutions per site.
(EPS)

Figure S4 **The PIG-A is involved in GPI anchor biosynthesis adding the first Glc-NAC to the phosphatidylinositol.** This activity could be missing in the analyzed Rhodophytes. The scale bar indicates the average number of amino acid substitutions per site.
(EPS)

Figure S5 **The ALG genes are involved in N-glycoprotein biosynthesis.** ALG2 and ALG11 add mannoses onto the growing glycan in the ER. Additional clades with unknown activity are also placed in the tree. Some branches are kinked to decrease the space requirements of the figure, the total branch length is still correct. The scale bar indicates the average number of amino acid substitutions per site.
(EPS)

Figure S6 **The starch synthases, both soluble and granular bound forms, are conserved among all the Viridiplantae analyzed.** Rhodophytes produce floridean starch, which is containing similar glycosydic linkages as starch from Viridiplantae, but of dissimilar structure. This is which is reflected in GT5 as the orthologous starch synthases found in red algae are quite divergent from the Viridiplantae starch synthases (not included in the tree). The scale bar indicates the average number of amino acid substitutions per site.
(EPS)

Figure S7 **The only GT8’s found in present study are orthologous to the PGSIP6, PGSIP7 and PGSIP 8 clades, respectively.** PGSIP6-8 have no reported activity. Both green and red algae have PGSIP6 orthologs whereas only the red algae have PGSIP7 and 8 orthologs. *S. moellendorffii* and *P. patens* are not included in the tree. The scale bar indicates the average number of amino acid substitutions per site.
(EPS)

Figure S8 **GT20 are trehalose synthases, and all analyzed genomes have orthologous proteins.** Some branches are kinked to decrease the space requirements of the figure, the total branch length is still correct. The scale bar indicates the average number of amino acid substitutions per site.
(EPS)

Figure S9 **Callose synthases are found in GT48. Only the Chlorophytes have GT48 members.** The domain structure is not fully conserved between plants and Chlorophytes. Some branches are kinked to decrease the space requirements of the figure, the total branch length is still correct. The scale bar indicates the average number of amino acid substitutions per site.
(EPS)

Figure S10 **ALG1 orthologs are found in GT33. ALG1 is a mannosyltransferase, adding the first mannose in N-glycan biosynthesis.** All species analyzed have ALG1 orthologs. Some branches are kinked to decrease the space requirements of the figure, the total branch length is still correct. The scale bar indicates the average number of amino acid substitutions per site.
(EPS)

Figure S11 **GT2 contain, among other activity, DPM and ALG5, which are mannosyl and glucosyltransferases, respectively.** They are using dolichol as acceptor and generate substrates for the mannosylation and glucosylation in N-glycan biosynthesis. Both activities are found in all analyzed genomes. In addition to these known activities, several algae sequences with unknown activity are observed. Some branches are kinked to decrease the space requirements of the figure, the total branch length is still correct. The scale bar indicates the average number of amino acid substitutions per site.
(EPS)

Figure S12 **ALG3 is a mannosyltransferase involved in N-glycan mannosylation.** Orthologs are found in the analyzed Rhodophyte and Prasinophyte genomes. Interestingly they were not observed in the analyzed core Chlorophyte genomes. The scale bar indicates the average number of amino acid substitutions per site.
(EPS)

Figure S13 **GT22 contain activities involved in N-glycan biosynthesis, ALG9 and ALG12, which are mannosyltransferases.** Both activites are missing in the core chlorophytes sequences analyzed. Beside the N-glycan biosynthetic activities, orthologs to PIGB and PIGZ which are GPI anchor biosynthetic GTs, can be found in GT22. With regards to the PIGB and PIGZ, it is remarkable that the core chlorophyte are missing PIGB and that the prasinophytes have PIGZ. PIGZ has to the authors knowledge not been found in Viridiplantae before. The scale bar indicates the average number of amino acid substitutions per site.
(EPS)

Figure S14 **The proteins found in GT57 are orthologous to ALG6, a glucosyltransferase involved in the first glucosylation and ALG8, the penultimate glucosylation of N-glycans in the ER.** The prasinophyte genomes analyzed do not have orthologous proteins. The *Micromonas* sp. ALG8 is a conundrum, since ALG6, which *Micromonas* sp. is missing, is a prerequisite for biosynthesis for ALG8 acceptor. The *Micromonas* sp. ALG8 could be a relic which *Micromonas* sp. have not lost yet. Some branches are kinked to decrease the space requirements of the figure, the total branch length is still correct. The scale bar indicates the average number of amino acid substitutions per site.
(EPS)

Figure S15 **GT10 contain complex N-glycan fucosyltransferases of which the core chlorophytes have orthologous proteins to the FUCTA.** FUCTA is responsible for the core β-1,3 fucosylation of the innermost GlcNAC. The scale bar indicates the average number of amino acid substitutions per site.
(EPS)

Figure S16 **The GlcNAC transferase activity of GTs found in GT41 catalyses the transfer of GlcNAC onto Ser or Thr.** Plant have both SPY and SEC orthologs, the same goes with *G. sulphuraria*, whereas the chlorophytes only contain SPY orthologs. The scale bar indicates the average number of amino acid substitutions per site.
(EPS)

Figure S17 **In GT75, the UDP-L-arabinopyranose mutase activity can be found.** Proteins with orthology to the plant GT75s could be found in *C. reinhardtii* and *V. carteri f. nagariensis*. The scale bar indicates the average number of amino acid substitutions per site.
(EPS)

Figure S18 **Orthologs to the GT47E clade were identified in the Chlorophyte genomes analyzed.** No activity has been published for this clade of GT47. The scale bar indicates the average number of amino acid substitutions per site.
(EPS)

Table S1 Overview of the genes found the analyzed genomes. For *C. reinhardtii* and *V. carteri f. nagariensis* the JGI protein ID is given as reference. For *O. tauri, O. lucimarinus* and *Micromonas* sp. the genbank accesion used as reference in CAZy is provided as reference. For *C. merolae* and *G. sulphuraria* the reference given is to the used public databases described in materials and methods. Genes that are not named are not included in the phylogenetic trees due to obvious and large mistakes, be it annotation mistakes or pseudogenes.
(XLSX)
